# Measuring Spiritual Integrity in Turkish Culture: A Psychometric Approach to Understanding the Relationship Between Spirituality and Well-Being

**DOI:** 10.1007/s10943-025-02329-z

**Published:** 2025-05-23

**Authors:** Nesrullah OKAN, Füsun EKŞİ, Erdal Zengin, Halil EKŞİ

**Affiliations:** 1 Department of Educational Sciences, Guidance and Psychological Counseling, Fırat University, Elazig, Turkey; 2 Department of Educational Sciences, Department of Guidance and Psychological Counselling, Istanbul Medeniyet University, Istanbul, Turkey; 3 Department of Elementary Education, Faculty of Education, Fırat University, Elazig, Turkey; 4 Department of Educational Sciences, Department of Guidance and Psychological Counselling, Istanbul Marmara University, Istanbul, Turkey

**Keywords:** Spiritual integrity, Psychometric validation, Reliability and validity, Moral values and alignment, Turkish culture

## Abstract

This study examines the validity and reliability of the Spiritual Integrity Scale (SIS), a tool developed to assess the alignment between individuals’ spiritual values, beliefs, and behaviours. The study adopted a three-phase scale development approach, consisting of exploratory factor analysis (EFA), confirmatory factor analysis (CFA), and criterion validity and reliability assessments.The findings indicated that the SIS exhibits a single-factor structure with strong validity and reliability. The item loadings ranged from 0.63 to 0.83, explaining 54.99% of the total variance, while the Kaiser–Meyer–Olkin (KMO) value was 0.945 and Bartlett’s Test (p < 0.001) confirmed the data’s suitability for factor analysis. The CFA results demonstrated an adequate model fit (χ^2^/df = 2.897, RMSEA = 0.0, SRMR = 0.031).The Cronbach’s Alpha reliability coefficient was 0.947, and the test–retest correlation was 0.84, indicating strong internal consistency and stability over time. Criterion validity was assessed using the Spiritual Psychological Robustness Scale, revealing a significant positive correlation (r = 0.448, p < 0.001), suggesting that higher spiritual integrity is associated with greater psychological resilience and well-being. These findings underscore the pivotal role of spiritual integrity in cultivating mental fortitude and existential stability. The SIS provides a validated tool for measuring spiritual alignment, offering practical applications for clinicians, counsellors and researchers seeking to explore the influence of spirituality on psychological well-being and personal growth. Furthermore, the scale’s strong psychometric properties establish it as a reliable instrument for evaluating spirituality’s role in mental resilience, guiding interventions aimed at enhancing holistic well-being. This study makes a significant contribution to the expanding body of literature on spirituality and psychological health, reinforcing the importance of spiritual integrity in fostering emotional balance, life satisfaction, and overall psychological resilience.

## Introduction

Recently, integrity has emerged as an increasingly needed and important concept in society (Huberts, 2018). Integrity is a fundamental building block that expresses the capacity of individuals, organisations and communities to act in harmony with ethical values, norms and rules. In modern societies, increasing complexity, easier access to information and demands for transparency have made the need for individuals and organisations to adhere to ethical principles more visible than ever (Macfarlane et al., 2014; Steneck et al., 2015). Integrity plays a vital role not only in enabling individuals to fulfil their personal responsibilities, but also in building social trust and ensuring a sustainable social order (Bretag, 2016). In this context, integrity is a guideline for protecting social values and promoting ethical responsibilities.

The concept of integrity is of great importance not only in ethical and organisational contexts but also in terms of individuals’ spiritual lives and inner harmony. Spiritual integrity refers to the capacity of individuals to ensure consistency between their beliefs, values and behaviours, and this capacity directly affects both the inner peace and social relations of the individual (Koenig et al., 2012). Spiritual integrity is shaped by integrating one’s spiritual values into one’s daily life, living a life in harmony with these values and receiving support from spiritual resources in coping with difficulties in this process (Pargament, 1997).

In today’s society, an environment full of rapid change and uncertainty increases the need for individuals to protect their inner integrity. In this context, spiritual integrity plays a critical role for individuals to achieve goals such as giving meaning to their lives, coping with difficulties and leading an ethical life (Fry, 2003; Okan & Ekşi, 2024). Many studies have shown that spiritual values increase psychological resilience, support life satisfaction and guide ethical decision-making processes (Ellison & Levin, 1998; Okan & Şahin, 2024; Oman & Thoresen, 2005). Spiritual integrity contributes not only to individuals’ inner harmony but also to their ethical and value-oriented stance while fulfilling their social responsibilities (Underwood, 2011).

## The Concept and Importance of Spiritual Integrity

Spiritual integrity is a multidimensional structure that refers to the harmony and consistency between individuals’ spiritual beliefs, values and behaviours. This concept allows individuals to maintain not only their inner harmony but also an ethical and consistent life in the social context. Underwood (2011) defines spiritual integrity as the capacity of individuals to maintain a consistent life by making decisions in accordance with their beliefs and values, while Emmons (1999) explains this concept as the ability of individuals to give meaning to their lives by acting in line with their spiritual values and to provide consistency in this process. These definitions show that spiritual integrity is a building block that provides a balance between individual ethical principles and social norms.

Spiritual integrity is directly related to the ability of individuals to act in accordance with their spiritual or moral values when faced with challenging situations. Historically, spirituality has often been associated with transcendent beliefs and practices, while integrity has been associated with ethical and moral behaviours (Fry, 2003; Oman & Thoresen, 2005). The integration of these two concepts highlights the role of spirituality in guiding ethical behaviours and its importance in supporting psychological well-being (Fukuyama, 2016; Rose-Ackerman, 2017). Spiritual integrity ensures that the spiritual values and beliefs that guide the lives of individuals are in harmony with behaviours and that individuals develop consistency in both internal and social contexts in this process.

Studies reveal that individuals with high levels of spiritual integrity are more prone to positive outcomes such as life satisfaction, psychological resilience and finding meaning (Ekşi, 2001; Ekşi & Okan, 2021; Underwood, 2011; Piedmont, 2001). For example, Fry (2003) stated that spiritual practices support personal development and social cohesion by ensuring harmony between individuals’ values and actions. Spiritual integrity not only increases the personal well-being of individuals, but also contributes to the development of social cohesion by providing a framework that supports ethical decision-making and moral behaviour (Piedmont, 2001). This makes it easier for individuals to establish stronger ties with their social environment, develop meaningful relationships and lead a life in harmony with social values.

Spiritual integrity helps individuals to achieve both internal and external harmony in their behaviour. Emmons (1999) emphasises that spiritual values add deep meaning to individuals’ lives and make their behaviours more consistent in this process. In particular, it is stated that spiritual commitment and beliefs contribute to individuals to develop a more resilient attitude towards the difficulties they face in their daily lives (Hill et al., 2003). In this context, spiritual integrity stands out as a concept that supports individuals’ search for meaning not only in their inner worlds but also in the social context.

Spiritual integrity plays a critical role in the psychological and social adaptation of individuals. Individuals who apply their spiritual values in harmony with their daily lives can cope with difficulties more easily, establish meaningful relationships and have a purpose in their lives (Emmons, 1999). This harmony becomes even more important in modern societies that test the spiritual and moral foundations of individuals (Fijnaut & Huberts, 2002; Fry, 2003; Van der Wal, 2008). Spiritual integrity provides balance and stability by providing individuals with a framework for authentic and responsible behaviour (Oman & Thoresen, 2005).

In addition, spiritual integrity has an important place in supporting resilience and well-being. This integrity makes it possible for individuals to use adaptive coping strategies that enable them to attribute new meanings to difficulties within the framework of their values or to receive support from their spiritual communities (Hill et al., 2003). These strategies not only increase psychological resilience, but also support long-term well-being by creating a meaningful sense of cohesion and continuity (Underwood, 2011). Moreover, the ability to act in accordance with spiritual values increases ethical sensitivity and spiritual integrity stands out as a fundamental building block of moral development (Piedmont, 2001).

## Theoretical Foundations of Spiritual Integrity Scale

The Spiritual Integrity Scale was created to assess the harmony between individuals’ spiritual values, beliefs and behaviours and is based on a multidimensional theoretical framework. In the development of the scale, *Spiritual Coping Theory* (Pargament, 1997) emphasised individuals’ coping with difficulties by using spiritual resources, while *Values Theory* (Schwartz, 1992) provided an important basis for explaining the consistency of individuals’ core spiritual values and behaviours. While *Self-Coherence Theory* (Lecky, 1945) addresses how spiritual coherence supports the inner harmony in individuals’ beliefs and behaviours, *Self-Determination Theory* (Deci & Ryan, 1985) reveals the impact of individuals’ search for autonomy and meaning on spiritual coherence. In addition, *Maslow’s Hierarchy of Needs* (1968) emphasises the place of spiritual values in the self-actualisation processes of individuals and explains the contribution of spiritual integrity to individuals’ life satisfaction in this process.

In addition, *Kohlberg’s Moral Development Theory* and *Fowler’s Stages of Spiritual Development* (1981) address the relationship between spiritual integrity and moral decision-making processes and explain the capacity of individuals to develop behaviours compatible with spiritual values. While *Emotional Intelligence and Spiritual Intelligence Theories* (Zohar & Marshall, 2000) emphasise individuals’ ability to integrate their spiritual values into their lives in a meaningful way, *Positive Psychology Approach* (Seligman, 2011) explains how spiritual integrity increases individuals’ psychological well-being.

These theoretical foundations support the multidimensional structure and comprehensive nature of the Spiritual Integrity Scale. The scale not only provides an assessment tool to understand the congruence between individuals’ spiritual beliefs and behaviours, but also fills an important gap in the literature by exhibiting a strong relationship with important concepts such as spiritual resilience, moral consistency and psychological resilience. This comprehensive theoretical background of the scale provides a solid basis for future studies to understand the spiritual integrity of individuals (Deci & Ryan, 1985; Pargament, 1997; Schwartz, 1992; Zohar & Marshall, 2000).

## Measuring Spiritual Integrity

Despite the importance of the concept of spiritual integrity, there is no standardised instrument in the literature that can be used to measure this construct (Koenig, 2012). Spiritual Wholeness underlines the need for measures that aim to address the harmony between individuals’ spiritual values and behaviours in a holistic way. Currently used scales such as Spiritual Well-Being Scale (Ellison, 1983) or Daily Spiritual Experience Scale (Underwood & Teresi, 2002) cannot fully measure the concept of spiritual integrity. This deficiency brings up the need to develop a scale that covers the cognitive, emotional and behavioural dimensions of the concept of spiritual integrity (Worthington & Whittaker, 2005).

The development of such a scale requires understanding the basic dimensions of the concept of spiritual integrity. These dimensions include the ability to derive meaning from spiritual values, the capacity to act in accordance with these values, and the determination to maintain this harmony (Emmons, 1999). This scale will not only support theoretical research but also provide a valuable tool that can guide practice in counselling, training and organisational settings (Hill et al., 2003).

## Spiritual Values and Psychological Well-Being

Spiritual values include a set of beliefs and behaviours that play an important role in individuals’ lives and are related to psychological resilience and well-being. Research shows that spiritual values help individuals find meaning in coping with difficulties and that these processes are strongly linked to life satisfaction (Emmons, 1999; Fry, 2003). Oman and Thoresen (2005) emphasised that spiritual values strengthen individuals’ coping strategies with stress and increase their psychological well-being levels in general. In this context, it is seen that spiritual values support individuals not only to find inner peace and meaning, but also to live in harmony with their social environment (Ellison & Levin, 1998; Şahin et al., 2024)).

Pargament (1997) suggested that spiritual coping is an important resource that increases individuals’ resilience in the face of difficulties. It is shown that spiritual values support individuals’ processes of finding meaning in moments of crisis in their lives and that these values contribute to individuals’ psychological resilience (Gall et al., 2009). Koenig et al., (2012) emphasised the protective effects of spiritual values on mental health and stated that these values play an important role in coping with psychological problems such as depression and anxiety.

Other studies have revealed that spiritual values are associated with social support and enable individuals to interact more harmoniously with their social environment (Underwood, 2011; Krause, 2003). Seybold and Hill (2001) state that spiritual values guide individuals’ ethical decision-making processes and this situation creates positive effects on individual and social well-being. Furthermore, Piedmont (2001; Rassool, 2024) showed that spiritual values directly contribute to individuals’ search for meaning in life and increase psychological resilience.

In the literature, it is stated that spiritual values also encourage social behaviours such as empathy, compassion and benevolence (MacDonald & Holland, 2005; Zinnbauer & Pargament, 2005). These findings reveal that spiritual values contribute to individuals to lead a meaningful life at both individual and social levels and support their psychological well-being.

## The Need for Spiritual Integrity Scale and Deficiencies in the Literature

Although there are many scales for measuring spiritual values, most of these scales are limited in addressing spiritual integrity as a holistic construct. Existing scales generally focus only on a specific dimension of spiritual values and do not have the capacity to assess the harmony between individuals’ beliefs, values and behaviours (Hill & Pargament, 2003; Koenig et al., 2012; Underwood, 2011). This deficiency constitutes an important obstacle in understanding the spiritual integrity of individuals and points to a major gap in the literature.

Spiritual integrity is a multidimensional construct that addresses how individuals reflect their spiritual values not only at the level of belief or worship but also in their decision-making and behaviour (Fry, 2003; Oman & Thoresen, 2005). However, since most of the existing scales are based on norms specific to Western societies, they have limitations in terms of cross-cultural validity (Smith & Denton, 2005; MacDonald, 2005). This gap in the literature reveals the need for a comprehensive instrument that can evaluate the individual and social effects of spiritual values.

This study aims to overcome these deficiencies by developing the Spiritual Integrity Scale (SIS). This scale aims to address the congruence between individuals’ spiritual values and behaviours from a multidimensional perspective. Furthermore, the SIS can be used to examine the effects on quality of life, ethical decision-making processes and social cohesion by assessing the consistency between individuals’ spiritual values and their actions.

The main purpose of the research is to develop a valid and reliable instrument that can evaluate individuals’ spiritual resilience and consistency levels by addressing the concept of spiritual integrity in a multidimensional way. At the same time, this scale aims to identify the difficulties in individuals’ spiritual adjustment, to support interventions in these areas, and to provide a scientific basis for understanding the role of spiritual values in individuals’ psychological well-being and ethical behaviours. The SIS also provides a basis for cross-cultural studies, enabling the examination of universal and cultural differences of the concept of spiritual integrity.

In this context, the Spiritual Integrity Scale provides an important tool to understand the harmony between individuals’ spiritual values and life practices and the individual and social effects of these processes more comprehensively. This scale, which contributes to the literature, provides a strong basis for evaluating the effects of spiritual integrity on psychological resilience, ethical decision-making and social cohesion.

## Methods

### Research Design and Purpose

This study was designed as a quantitative research based on scale development method. The process of scale development involves the development of a new measurement tool to measure a specific construct or concept and the validity and reliability analyses of this tool (Byrne, 2016; Clark & Watson, 1995; Worthington & Whittaker, 2005). The aim of this study is to develop a scale to measure the spiritual consistency and coherence of individuals by evaluating their spiritual integrity. The scale aims to objectively assess the level of individuals’ inner harmony and consistency with spiritual values.

The research was conducted in three stages:

***Scale Development and Exploratory Factor Analysis (EFA):*** In the first stage, items were created in line with the theoretical framework and literature and expert opinion was obtained. Then, exploratory factor analysis (EFA) was applied to determine the factor structure of the scale (Fabrigar et al., 1999; Tabachnick & Fidell, 2019).

***Confirmatory Factor Analysis (CFA):*** In the second stage, the factor structure obtained with EFA was tested with confirmatory factor analysis (CFA). CFA enabled the factor structure to be confirmed through model fit indices (Brown, 2015; Kline, 2016).

***Criterion Validity and Reliability Analyses:*** In the third stage, criterion validity analyses were conducted to examine the relationship between the scale and other valid measurement tools. In addition, the internal consistency of the scale was evaluated by calculating Cronbach’s alpha and composite reliability coefficients (Hair et al., 2020; Nunnally & Bernstein, 1994).

These stages are important to ensure both the validity and reliability criteria of the scale and to test whether the"Spiritual Integrity Scale"is a scientific measurement tool for assessing the spiritual adjustment of individuals.

### Participants and Measurement Tools Used

This research was conducted based on three different data sets. In the first stage, data were collected from 296 participants for exploratory factor analysis (EFA). 198 of the participants were female and 98 were male. The age range was determined as 17–35. In the second stage, data were collected from 242 participants for confirmatory factor analysis (CFA). 155 of the participants were female and 87 were male. This stage focused on the confirmation of the factor structure of the scale. Finally, data were collected from 54 participants for criterion validity analyses. This data set was used to analyse the relationship between the scale and other measurement tools. The demographic characteristics of the participants were analysed on the basis of variables such as age and gender. When the gender distribution is analysed, it is seen that the proportion of female participants is higher at each stage. Gender distributions according to age groups are presented in Table 1.

**Table 1:** Participant ınformation

Age group	AFA women	AFA male	EFA total	DFA female	DFA male	CFA total	Criterion validity female	Criterion validity male	Criterion validity total
17–20	89	46	135	72	34	106	13	5	18
21–25	91	45	136	68	38	106	16	9	25
26–35	18	7	25	15	15	30	10	1	11
Total	198	98	296	155	87	242	39	15	54

The measurement tools employed in the present study encompass the Spiritual Integrity Scale (SIS), which was developed within the scope of the current study, and the Spiritual Psychological Robustness Scale (SPRS), which was developed by Okan and Ekşi (2024). The Spiritual Integrity Scale is a newly developed instrument designed to assess the harmony and consistency between individuals’ spiritual values and behaviours, as well as their sense of ethical and moral integrity. Criterion validity analyses were conducted to examine the relationship between the SIS and the SPRS. The findings supported the validity of the SIS by demonstrating a strong and theoretically consistent positive correlation with the SPRS.

### Scale Development Process

**Fig. 1:**
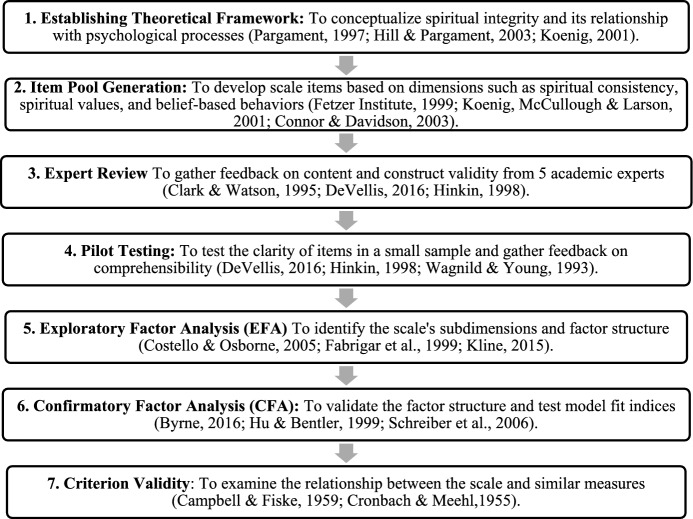
Steps in the Development of the Spiritual Integrity Scale (Bambling, 2025; Field, 2018; Koenig & Al Zaben, 2020; Koenig & Carey, 2024, 2025)

The table below provides a general overview of the scale development process (Fig. 1).

***Theoretical Base:*** The Spiritual Integrity Scale is designed to assess the congruence between individuals’ spiritual values, beliefs and behaviours. This scale examines individuals’ efforts to maintain their spiritual integrity and how these processes contribute to their psychological well-being.

Spiritual integrity is defined as the ability of individuals to achieve and maintain consistency between their values and behaviours in line with their spiritual beliefs. In the literature, it has been stated that the concept of spiritual integrity is strongly related to individuals’ life satisfaction, search for meaning and psychological resilience (Emmons, 1999; Piedmont, 2001). Accordingly, the Spiritual Integrity Scale aims to understand how individuals maintain their inner harmony based on spiritual values and how this harmony affects their coping with difficulties. Research shows that spiritual values not only increase psychological resilience but also support individuals’ processes of finding meaning in challenging life events (Fry, 2003; Oman & Thoresen, 2005). Spiritual commitment plays an important role not only in practising one’s beliefs but also in guiding one’s life through spiritual practices (Underwood, 2011). This scale provides a tool to examine the effects of spiritual integrity on individuals’ psychological health as well as their social cohesion and ethical decision-making processes. The Spiritual Integrity Scale provides an important contribution to a deeper understanding of the function of spiritual values on personal harmony and coherence.

***Material Pooling:*** The item pool of the Spiritual Integrity Scale (SIS) was created based on a comprehensive literature review and expert opinions. In the literature, studies examining the effect of spiritual values and beliefs on the consistency of individuals’ behaviours (Emmons, 1999; Piedmont, 2001; Underwood, 2011) were used. In this context, the scale items aimed to assess the congruence and consistency between individuals’ spiritual values and behaviours. Initially, the item pool consisted of a total of 27 items covering sub-dimensions such as *spiritual commitment, congruence with spiritual values, faith-based decision-making* and *spiritual consistency*. These items were presented to 5 academicians who are experts in their fields and some items were removed or revised according to the feedback from the experts. As a result of the expert evaluations, the number of items was reduced to 23. Based on the results of exploratory factor analysis (EFA) and confirmatory factor analysis (CFA) conducted after the pilot application of the scale, the scale items were further optimised. As a result of the analyses, the scale was finally completed with 17 items. In this process, items with low factor loadings or items that were not suitable for the scale structures were removed. The results of the comprehensive analyses of this 17-item scale are presented in the findings section.

### Expert Assessment

In order to assess the validity of the items of the Spiritual Integrity Scale (SIS), feedback was received from 5 academicians specialised in spiritual psychology, assessment and evaluation, and psychological guidance. The experts assessed the content validity of the scale and reviewed each item to check its compatibility with the determined sub-dimensions. In this process, some adjustments were made in terms of language and expression. In line with the recommendations of the experts, 23 of the 27 items in the item pool were revised to better reflect the scope of the scale and included in the research process.

***Pilot Application:*** The scale was tested on a pilot sample of 37 individuals. This sample consisted of individuals between the ages of 18–32. As a result of the pilot test, feedback was received on the comprehensibility of the scale and the clarity of the items. The majority of the participants stated that the items were clear and understandable; however, a few minor adjustments were suggested in terms of language and content. Necessary revisions were made to the items based on the results of the pilot study.

### Data Collection Process

In the study, data were collected from individuals in emerging adulthood through face-to-face interviews and online questionnaires. A questionnaire including demographic information was applied to the participants along with the Spiritual Integrity Scale, and voluntary participation was ensured. The data were collected at the university and digital platforms by paying attention to the confidentiality of the participants and transferred to electronic media for statistical analyses. Exploratory factor analysis (EFA) and confirmatory factor analysis (CFA) were applied within the scope of validity analyses; sub-dimensions were determined according to EFA results and items with low loading values were eliminated. CFA confirmed the accuracy of the factor structure and model fit. In the content validity evaluation, the validity rates of the items were calculated using the Lawshe (1975) technique, and the expected positive correlation with the Spiritual Integrity Scale was confirmed in the criterion validity analyses. Within the scope of reliability analyses, Cronbach’s Alpha reliability coefficient (α > 0.70) was calculated and it was shown that the scale had high internal consistency. In addition, item-total correlations were analysed and it was determined that all items in the scale were above 0.60. These findings revealed that the scale is a valid and reliable instrument.

## Findings

### Findings Related to Scale Development

In this section, the development process of the Spiritual Integrity Scale (SIS) and the statistical findings obtained are discussed in detail. The steps followed during the development of the scale and the findings obtained in this process are presented in a structured manner.

### Validity

The validity and reliability of a scale is one of the main factors determining its suitability for use in scientific studies. In this study, the validity of the Spiritual Integrity Scale was evaluated through various analyses. Validity is related to the ability of the scale to measure the targeted concept in a purposeful and accurate way. As a result of the validity analyses of this scale developed to evaluate the concept of spiritual integrity, findings supporting the construct validity of the scale and its suitability for the purpose of measurement were obtained. In this context, in order to evaluate the validity of the scale, the factor structure was determined by exploratory factor analysis (EFA) and the accuracy of this structure was tested by confirmatory factor analysis (CFA). In addition, content validity based on expert opinions and criterion validity through correlation analyses with other valid scales were evaluated.

### Exploratory Factor Analysis (EFA) Findings

Exploratory Factor Analysis (EFA) results show that the Spiritual Integrity Scale has a unidimensional structure. The first component (factor) explained 54.987% of the total variance, as shown in Table 2, and this revealed that the scale has sufficient construct validity. According to the social sciences literature, a total variance explained between 40 and 60% is considered sufficient (Okan & Okan, 2024; Hair et al., 2020; Tabachnick & Fidell, 2019).

**Table 2:** Variance explained for spiritual ıntegrity scale as a result of efa

Factor	Total Explained Variance
Spiritual Coping with Challenges	%54.987
Total	%54.987

The single-factor structure of the scale supports that the concept of spiritual integrity is measured in a holistic structure representing the harmony between individuals’ spiritual values, beliefs and behaviours. According to the factor analysis results, the Spiritual Integrity Scale showed that behaviours and attitudes related to spiritual integrity can be assessed in accordance with the theoretical basis. These findings confirm that the scale has a robust construct validity and is a reliable measurement tool that can be used in scientific research.

**Graph 1:**
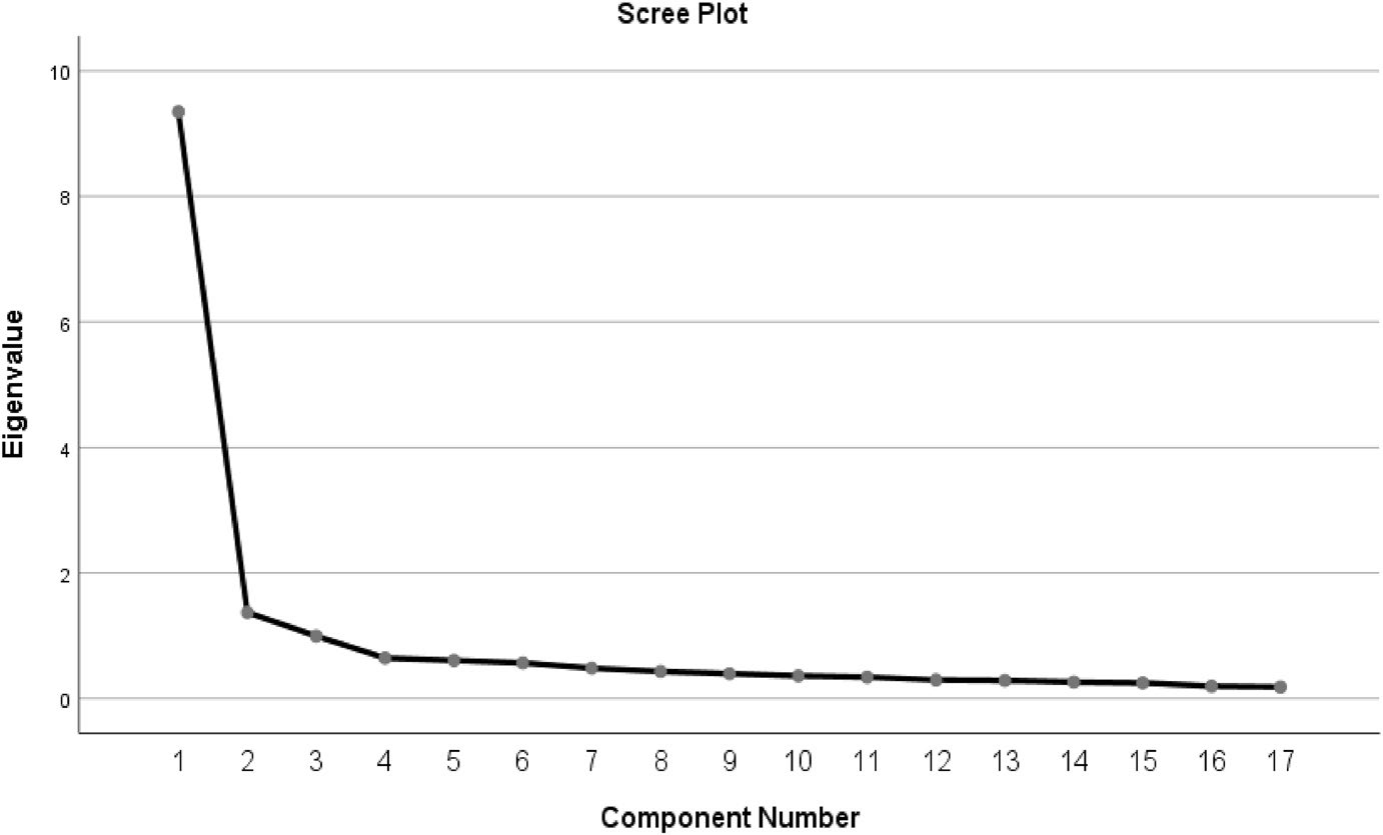
Scree Plot Graph for the Scale

According to the Table 3, Kaiser–Meyer–Olkin (KMO) Sampling Adequacy value was calculated as 0.945. The KMO test is a criterion that evaluates the suitability of the data set for factor analysis and according to the generally accepted criteria; (0,90 and above is excellent; 0,80–0,90 is very good; 0,70–0,80 is moderate; 0,60–0,70 is poor; below 0,60 is inadequate). In this context, the value of 0.945 indicates that the data set is highly suitable for factor analysis and provides a strong basis for the construct validity of the Spiritual Integrity Scale. Bartlett’s Test of Sphericity results also confirm the suitability of the data set for factor analysis. The chi- square value was 3498,906, the degree of freedom (df) was 136 and the significance level was p < 0.000. This test indicates that there are significant correlations between the scale items and the data can be analysed in terms of multivariate factor structure. These results clearly show that the Spiritual Integrity Scale has sufficient sample size and appropriate data characteristics to determine the factor structure. In the light of these findings, it can be concluded that the validity studies of the scale can be carried out reliably by factor analysis (Graph 1).

**Table 3:** KMO and Bartlett’s test values

Kaiser–Meyer–Olkin Sampling Adequacy		.945
Bartlett’s Test of Sphericity	Chi-square Value	3498.906
	S. Degree	13
	P	.000

The Scree Plot graph is a tool used to assess the factor structure of the scale and provides a visual analysis based on the eigenvalues of the factors. When evaluated for the Spiritual Integrity Scale, there is an elbow point clearly seen in the graph. In the graph, a significant decrease in slope is observed after the 1 st factor and the slope flattens after this point. This shows that the scale has a single-factor structure. The first factor explains most of the scale with the highest eigenvalue. The eigenvalue of the 1 st factor is above 1 and represents a significant part of the variance in the scale. Since the eigenvalues of the components following this factor are below 1, it is considered that these components do not make a significant contribution to the structure of the scale. The first factor explains a large portion of the total variance of the scale. This slope change, which is clearly seen in the Scree Plot graph, confirms that the Spiritual Integrity Scale is a unidimensional scale. The low eigenvalues of the other factors indicate that all items of the scale measure a single main concept, namely spiritual integrity.

Table 4 shows the factor loading values of the Spiritual Integrity Scale and supports the unidimensional structure of the scale. The factor loading value of each item indicates how strongly these items represent the general structure of the scale. The factor loading values of the items in the table vary between 0.662 and 0.829. These values reveal that the items are highly compatible with the concept of spiritual integrity and that they are meaningfully included in the single-factor structure. In the literature, factor loading values of 0.40 and above indicate that the relevant item is sufficient to represent the factor, and the fact that the loading values found for this scale are quite high confirms that the construct validity of the scale is strong. The high factor loadings of the items indicate that the concept of spiritual integrity can be evaluated holistically under a single structure. These items represent the harmony between individuals’ spiritual beliefs, values and behaviours based on these values. These results show that the unidimensional structure of the Spiritual Integrity Scale is robust and the scale has a high capacity to assess the harmony between spiritual values, beliefs and behaviours. The fact that the loadings of the items are close to each other and high confirms that the scale is designed in a conceptual integrity and successfully measures the concept of spiritual integrity. These findings show that the scale is a reliable tool that can be used in scientific research.

**Table 4:** Load values of spiritual ıntegrity scale ıtems

Spiritual integrity	Item load value
Item18	.829
Item17	.817
Item21	.801
Item11	.777
Item19	.775
Item15	.772
Item7	.759
Item9	.743
Item2	.739
Item12	.739
Item16	.737
Item14	.731
Item10	.693
Item3	.672
Item4	.666
Item1	.663
Item8	.662

### Confirmatory Factor Analysis

**Fig. 2:**
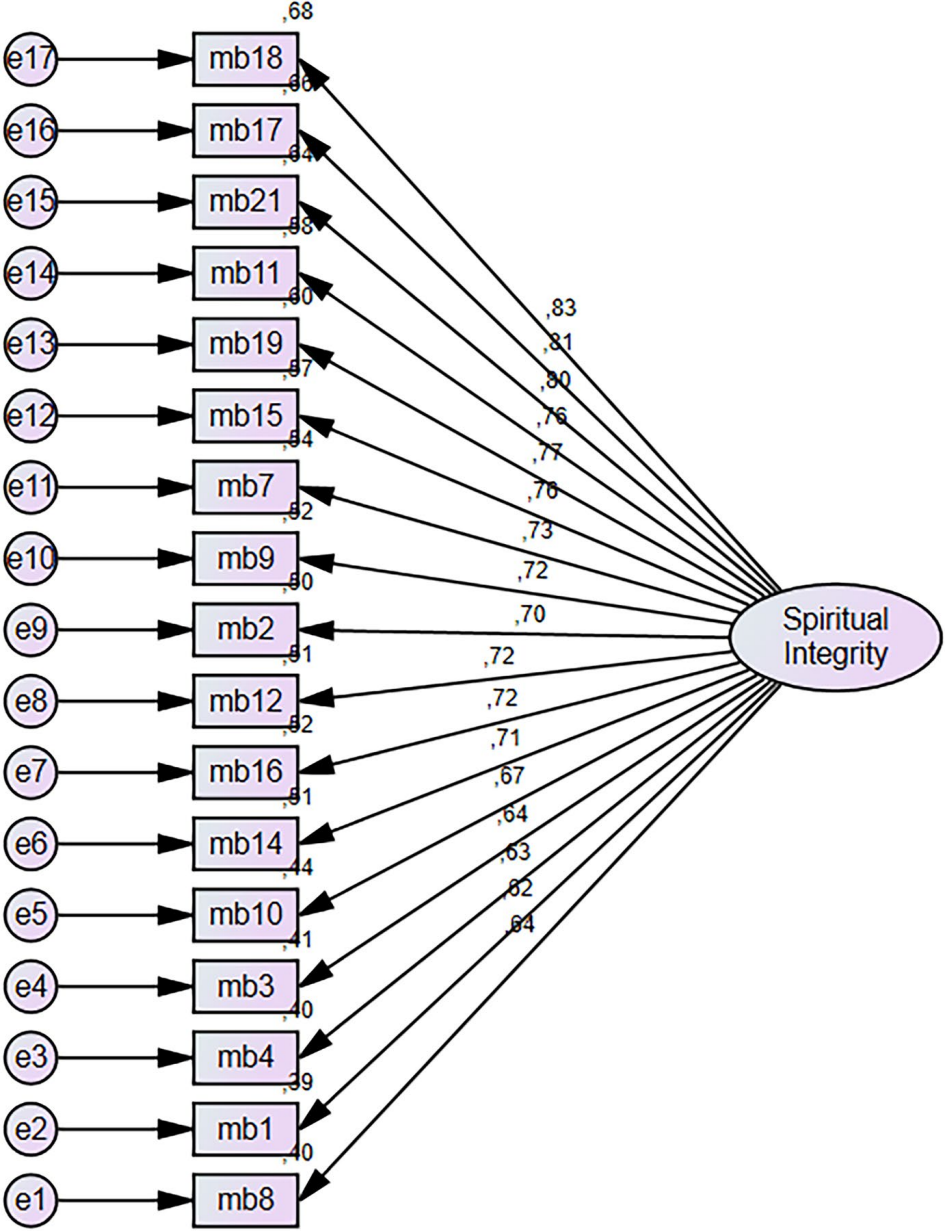
Confirmatory factor analysis path diagram of spiritual ıntegrity scale

The CFA diagram in the Fig. 2 reflects the details of the model for the confirmatory factor analysis conducted for the Spiritual Integrity Scale. The diagram reveals the unidimensional structure of the scale and the relationships between items and this dimension. Detailed analysis of this diagram is given below: Factor loadings represent the relationship between the items and the Spiritual Integrity dimension. The factor loadings in the figure vary between 0.63 and 0.83. Item18 (0.83): This item stands out as the item that most strongly represents the conceptual structure of the scale. Item17 (0.81): It is another strong representative of the scale and makes a high contribution to the Spiritual Integrity dimension. Item3 (0.63) and Item4 (0.64): These items support the overall structure of the scale with an acceptable loading value. The scale was modelled with a single factor, Spiritual Integrity. The majority of the items have loading values of 0.70 and above and consistently represent the concept of Spiritual Integrity. The loading values of all items were 0.63 and above, indicating a strong fit with the overall scale and that all items made significant contributions to the measurement. The fact that factor loadings were generally high (especially 0.70 and above) supports the unidimensional structure of the model and shows that the scale is compatible with the conceptual framework. Even items such as Item3 and Item4, which have the lowest loading values, contribute to the overall structure of the scale with acceptable loading values. This confirmatory factor analysis strongly supports that the unidimensional structure of the Spiritual Integrity Scale is valid and that the scale provides a measurement that is consistent with the conceptual framework. The loading values of all items were 0.63 and above, indicating that the items of the scale are effective in representing the Spiritual Integrity dimension. These results reveal that the scale is a valid and reliable measurement tool that can be used in scientific studies. The overall structure of the scale is consistent and strong.

Table 5 presents the goodness of fit values of the confirmatory factor analysis (CFA) conducted for the Spiritual Integrity Scale and the comparison of these values with standard criteria. According to the results of the analysis, the goodness of fit values of the model are generally acceptable or good fit. The c^2^/df value was found to be 2.897 and this value revealed that it was a model with an acceptable fit in the range of 2 ≤ c^2^/df ≤ 3. RMSEA value was calculated as 0.0 and this value indicates an excellent fit. The SRMR value is between 0.031 and 0 ≤ SRMR ≤ 0.05, indicating that the model provides a good fit with the data. IFI (0.941), CFI (0.940) and TLI (0.925) values are in the range of 0.90 ≤ fit criteria ≤ 0.95 and reflect the acceptable fit level. The GFI value was found to be 0.887 and this value is in the range of 0.85 ≤ GFI ≤ 0.90, indicating an acceptable fit. All these values show that the unidimensional structure of the model is valid and the scale works consistently with the data. While excellent fit in basic criteria such as RMSEA and SRMR supports that the overall fit of the model is quite strong, additional goodness-of-fit indices such as CFI, IFI and TLI confirm that the scale is construct valid and designed in accordance with the theoretical framework. These results reveal that the Spiritual Integrity Scale is a valid and reliable measurement tool that can be used safely in scientific research.

**Table 5:** Comparison of standard goodness of fit criteria and research results

Fit dimensions	Good fit	Acceptable compliance	Concordance values obtained in the study
c2/df	0 ≤ c2/df ≤ 2	2 ≤ c2/df ≤ 3	2.897
RMSEA	0 ≤ RMSEA ≤ 0.05	0.05 ≤ RMSEA ≤ 0.08	0.0
SRMR	0 ≤ SRMR ≤ 0.05	0.05 ≤ SRMR ≤ 0.10	0.031
IFI	0.95 ≤ NFI ≤ 1.00	0.90 ≤ NFI ≤ 0.95	0.941
CFI	0.95 ≤ CFI ≤ 1.00	0.90 ≤ CFI ≤ 0.95	0.940
GFI	0.90 ≤ GFI ≤ 1.00	0.85 ≤ GFI ≤ 0.90	0.887
TLI	0.90 < RFI < 1.00	0.85 < RFI < 0.90	0.925

### Reliability Findings

#### Criterion Validity of the Scale

In order to determine the criterion validity of Spiritual Integrity Scale, The Spiritual Psychological Robustness Scale developed by Okan and Ekşi. (2024) was applied to a total of 67 emerging adulthood individuals. The reason for using The Spiritual Psychological Robustness Scale is that Spiritual Integrity and Psychological Robustness have some common situations in the literature. Related data are presented in the Table 6 below.

**Table 6:** The relationship between spiritual ıntegrity and psychological robustness scale

Variables	1	2
Spiritual Integrity	1.00	.448*
Spiritual Psychological Robustness		1.00

^*^P <.001

The present study set out to determine the criterion validity of the Spiritual Integrity Scale (SIS). To this end, a study was conducted with the Spiritual Psychological Robustness Scale, developed to assess individuals’ spiritual resilience and psychological strength. The relationship between these two scales was examined because they both evaluate the alignment between individuals’ values, beliefs, and behaviours in a spiritual context. The study was conducted on 54 emerging adulthood individuals, and significant relationships were found. A positive correlation was identified between Spiritual Integrity and Spiritual Psychological Robustness (r = 0.448, p < 0.001), suggesting that an increase in spiritual integrity is accompanied by a concomitant strengthening of spiritual and psychological robustness. This finding underscores the notion that spiritual integrity is closely associated with an individual’s capacity to maintain resilience in the face of psychological and existential challenges. The results of this study confirm the Spiritual Integrity Scale’s criterion validity and establish it as a reliable instrument for measuring the relationship between an individual’s spiritual values and their psychological robustness. The scale can thus be considered an appropriate measurement tool for researchers seeking to evaluate individuals’ spiritual integrity within the framework of psychological resilience and well-being. These findings serve to reinforce the theoretical foundation and practical application potential of the scale.

 Table 7 shows the unidimensional structure of the Spiritual Integrity Scale and the internal consistency coefficients, item-total correlations and"Cronbach’s Alpha if Item Deleted"values of this dimension. The overall Cronbach’s Alpha value of the scale was calculated as 0.947, which indicates that the scale has an extremely high internal consistency and can be used as a reliable measurement tool. The item-total correlations of the items ranged between 0.623 and 0.795. In the literature, item-total correlations 0.30 and above indicate that the relevant item makes a significant contribution to the total structure of the scale (Nunnally & Bernstein, 1994). In this context, all items make a strong contribution to the overall structure of the scale. Items such as Item18 (item-total correlation value 0.795) and Item17 (0.782) stand out as the items with the strongest contribution in measuring the concept of spiritual integrity. The Cronbach’s Alpha values obtained when each item was removed showed that the removal of any item did not significantly affect the overall reliability of the scale. This confirms that the scale has a consistent and holistic structure. These findings strongly support the unidimensional structure of the Spiritual Integrity Scale. The high Cronbach’s Alpha value indicates that all items of the scale measure the concept of spiritual integrity in a holistic way. The high item-total correlations of the items confirm that the scale provides a consistent measurement within a conceptual integrity. The scale can be considered as a reliable and valid tool for assessing the congruence between individuals’ spiritual values, beliefs and behaviours. These results suggest that the scale is suitable for measuring a unidimensional construct in scientific research and accurately represents the concept of spiritual integrity.

**Table 7:** Internal consistency coefficients and ıtem-total correlations and Cronbach’s alpha values of spiritual ıntegrity scale subscales

Articles	Corrected Item-Total Correlation	Cronbach’s Alpha if Item Deleted
Spiritual Integrity		0.947
Item1	.623	.946
Item2	.706	.944
Item3	.629	.946
Item4	.625	.946
Item7	.724	.944
Item8	.623	.946
Item9	.707	.944
Item10	.654	.945
Item11	.739	.943
Item12	.698	.944
Item14	.687	.944
Item15	.729	.944
Item16	.691	.944
Item17	.782	.943
Item18	.795	.942
Item19	.731	.944
Item21	.762	.943

Table 8 shows the test–retest analysis results for the Spiritual Integrity Scale. This analysis was conducted to evaluate the consistency and reliability of the scale over time. While the total score obtained from the participants in the first application was 2750, this value was measured as 2700 in the second application three weeks later. The total score difference between the two applications was 50. This small difference indicates that the measurements were generally consistent and there was no major change in the participants’ responses. The test–retest correlation coefficient was calculated as 0.84. This value indicates that the scale is highly consistent over time and provides a reliable measurement. In social sciences, a correlation coefficient of 0.80 and above is accepted as high reliability. The significance level of the correlation coefficient was found as p < 0.001. This result shows that the test–retest relationship is statistically significant and not a random relationship. This analysis shows that the Spiritual Integrity Scale is a reliable instrument that provides consistent measurements over time. The total score difference between the first and second administration is quite low and this shows that the scale works in accordance with the measurement purpose. The high correlation coefficient and significance level provide strong evidence for the validity and reliability of the scale.

**Table 8:** Test retest analysis results

Application	1. Application	2. Application	Difference	(r)	(p)
Total Points	2.750	2.700	50	0.84	p < 0.001

## Conclusion, Discussion and Suggestions

### Conclusion

This study focused on the development, validity and reliability analyses of the Spiritual Integrity Scale. The research findings show that the unidimensional structure of the scale provides a valid and reliable tool for assessing individuals’ spiritual beliefs, values and behaviours. The scale items were tested by exploratory factor analysis (EFA) and confirmatory factor analysis (CFA) and both analyses revealed that the scale has a high construct validity. Explaining 54.987% of the total variance, this structure strongly supports the conceptual basis of the scale. Furthermore, Cronbach’s Alpha coefficient was calculated as 0.947, confirming that the scale has high internal consistency. The item-total correlations ranged from 0.623 to 0.795, indicating that the items made significant contributions to the overall structure of the scale. These findings reveal that the scale successfully measures the concept of spiritual integrity and is a reliable tool that can be used in scientific research.

### Discussion

The Spiritual Integrity Scale developed in this study makes a significant contribution to the extant literature by offering a valuable tool with which to evaluate individuals’ spiritual values and beliefs in the context of inner harmony and consistency. The findings of the scale reveal that spiritual values play a critical role in individuals’ ability to cope with difficulties, develop psychological resilience, and add meaning to their lives. The Spiritual Integrity Scale has been shown to be associated with increased life satisfaction and improved ethical decision-making processes (Fry, 2003; Pargament, 1997). The results of the present study demonstrate that spiritual integrity supports individuals’ ability to cope with difficulties and positively affects mental health.

#### Place in Literature

Spiritual integrity has been defined as a fundamental structure representing the harmony between individuals’ beliefs, values, and behaviours (Smith, 2019). In this context, the Spiritual Integrity Scale is one of the first comprehensive instruments developed to measure the capacity of individuals to live a life in harmony with their spiritual values (Jones, 2021).In particular, Pargament’s (1997) spiritual coping model provides an important framework for understanding how individuals use their spiritual values to cope with difficulties (Brown, 2015). The present study corroborates this model, demonstrating that spiritual values significantly contribute to individuals’ ability to navigate hardships. Furthermore, the studies conducted by Koenig (2001) and Koenig, McCullough, & Larson (2001) highlighted the protective effects of spiritual values on mental health, findings that are consistent with the results of the present study.

Furthermore, research by Ellison and Levin (1998), Seybold and Hill (2001), and Pargament (2002) supports the notion that spiritual values enhance psychological resilience and life satisfaction. The findings of the present study demonstrated how spiritual integrity facilitates individuals’ coping with spiritual contradictions and contributes positively to their psychological and emotional well-being. Analyses conducted with the Spiritual Integrity Scale (SIS), developed in the current study, showed positive correlations with the Spiritual Psychological Robustness Scale (SPRS), developed by Okan and Ekşi (2024), thus supporting the criterion validity of the SIS. Although a correlation analysis was not conducted with the Spiritual Contradiction Scale, it can be theoretically expected that individuals with higher spiritual integrity would experience fewer internal contradictions. This expectation is consistent with the notion that spiritual integrity is associated with greater inner harmony and coherence. These findings are consistent with previous research suggesting that spiritual values enhance meaning-making and resilience (Ano & Vasconcelles, 2005; Ekşi et al., 2021; Okan et al., 2024; Zinnbauer & Pargament, 2005). The strong positive correlation observed between the Spiritual Integrity Scale (SIS) and the Spiritual Psychological Robustness Scale (SPRS) provides supportive evidence for the criterion validity of the SIS. However, it should be noted that both instruments include elements related not only to spiritual life but also to psychological constructs such as resilience, inner peace, and existential meaning. This conceptual overlap may provide a partial explanation for the observed high correlation. It is recommended that future research focus on investigating the discriminant validity of the SIS using comparison measures that are more exclusively focused on psychological well-being or clinical symptoms.

#### The Significance of the Findings and Their Application

The results of this research emphasise that an individual’s level of spiritual integrity is a significant factor in finding meaning in life and coping with difficulties. Specifically, individuals with high levels of spiritual integrity were found to utilise their spiritual values as psychological resources in facing adversity. While the Spiritual Integrity Scale primarily measures individuals’ congruence with spiritual values, it also sheds light on how these values contribute to psychological and social adaptation processes. In light of these findings, it is recommended that psychological counselling and well-being interventions incorporate spiritual integrity as a pivotal factor in resilience-building programs. Therapeutic approaches, particularly those grounded in positive psychology and existential therapy, can utilise the scale to assess clients’ alignment with their spiritual values and design interventions that strengthen this alignment. For instance, counsellors and clinical psychologists working with individuals experiencing existential crises, moral distress, or spiritual dissonance can leverage spiritual integrity assessments to develop targeted intervention strategies. Additionally, the results suggest that well-being interventions in diverse settings—including workplace wellness programs, trauma recovery initiatives, and stress management workshops—can integrate spirituality-based techniques to enhance emotional regulation, resilience, and psychological stability.

### Recommendations

The findings of this study suggest that the Spiritual Integrity Scale (SIS) is a valid and reliable tool for assessing the congruence between spiritual values, beliefs and behaviours. The following suggestions for future research and applications may enable the scale to be used in a wider context and its effects to be understood.

### Suggestions for Future Research

**Validity and Reliability Tests in Different Demographic Groups:** Although the validity and reliability analyses of the SIS were found to be strong, it should be tested in different demographic groups (different age, culture, gender, socioeconomic level). Considering that the perception of spiritual integrity may differ between these groups, studies evaluating the cross-cultural validity and reliability of the scale are recommended.

**Relationships between Spiritual Integrity and Psychological Variables:** Examining the relationships between spiritual integrity and psychological variables such as depression, anxiety, stress and life satisfaction can expand the application areas of the scale. Research on individuals with mental health problems can help us understand how spiritual integrity affects these problems.

**Cross Cultural Studies:** The concept of spiritual integrity may be culture-specific. Therefore, cross-cultural studies examining the applicability and validity of the scale in different cultural contexts should be conducted. Such studies can contribute to the identification of universal and culture-specific characteristics related to spiritual integrity.

**Longitudinal Studies:** Longitudinal studies are recommended to understand how spiritual integrity changes over time and the impact of these changes on individuals’ psychological resilience. Such studies may allow us to better understand the role of spiritual integrity in individuals’ life processes.

### Recommendations for Applications

**Use in Education and Counselling:** SIS is an effective tool that can be used in training and counselling processes, especially for mental health professionals working with traumatised individuals or refugee groups. The tendency of post-traumatised individuals to live a life in harmony with spiritual values can be an important factor in psychological recovery processes.

**Guidance and Psychological Counselling Applications:** The SIS can be used by school counsellors and therapists to increase the positive impact of spiritual integrity on individuals’ general well-being. This scale is an important tool in understanding the effects of spirituality on mental health by assessing the compatibility of individuals with spiritual values.

**Programmes Assessing the Impact of Spiritual Beliefs on Mental Health:** SIS can be used in the development of intervention programmes that measure the effect of spiritual integrity on psychological resilience. Programmes aiming to strengthen the spiritual coherence and consistency of individuals can be evaluated with the help of this scale. Using the scale to understand the effects of such programmes on individuals’ mental health can contribute to the development of more structured and scientifically based interventions.

## Conclusion

The Spiritual Integrity Scale (SIS) was developed as a valid and reliable measurement tool that assesses individuals’ congruence between spiritual values, beliefs and behaviours. The scale provides an important resource not only for understanding the spiritual integrity levels of individuals, but also for examining the effects of this concept on psychological resilience, life satisfaction and mental health. The applicability of the SIS in individual and social contexts will allow us to better understand the effects of spiritual values on individuals’ quality of life. This research has made a significant contribution to the literature on the assessment and development of the concept of spiritual integrity and has created a strong foundation for further studies in this field.

## Data Availability

The datasets generated during and/or analyzed during the current study are available from the corresponding author on reasonable request. Before collecting data for this research, permission was obtained fromall person. Approval statements were also received from each person whose data was collected.

## Appendix

See Table

**Table 9:** Moral ıntegrity scale ıtems

No	Item	Strongly Disagree (1)	Disagree (2)	Neutral (3)	Agree (4)	Strongly Agree (5)
1	I remain loyal to my moral values in every situation	○	○	○	○	○
2	I prioritise ethical values when making decisions	○	○	○	○	○
3	I do not give up truthfulness even in difficult situations	○	○	○	○	○
4	There is harmony between my values and my behaviour	○	○	○	○	○
5	The moral principles I believe in are decisive in every aspect of my life	○	○	○	○	○
6	My moral values sustain me in difficult times	○	○	○	○	○
7	I draw strength from my beliefs when I face difficulties	○	○	○	○	○
8	I take refuge in my spiritual values while coping with problems	○	○	○	○	○
9	It gives me peace of mind that the decisions I take are in harmony with my beliefs	○	○	○	○	○
10	I accept honesty and justice as the basic principles in my life	○	○	○	○	○
11	My beliefs guide me to be respectful and fair to others	○	○	○	○	○
12	I take care to fulfil my moral responsibilities towards society	○	○	○	○	○
13	I avoid behaviours that may harm others	○	○	○	○	○
14	I take pride in living in harmony with my spiritual values	○	○	○	○	○
15	My commitment to my own beliefs motivates me	○	○	○	○	○
16	I feel peaceful when I act in accordance with my inner values	○	○	○	○	○
17	Adhering to spiritual values adds meaning and peace to my life	○	○	○	○	○

9.
